# Cohort study of the effect of surgical repair of symptomatic diastasis recti abdominis on abdominal trunk function and quality of life

**DOI:** 10.1002/bjs5.50213

**Published:** 2019-09-11

**Authors:** A. Olsson, O. Kiwanuka, S. Wilhelmsson, G. Sandblom, O. Stackelberg

**Affiliations:** ^1^ Department of Clinical Science and Education Karolinska Institutet, Södersjukhuset Stockholm Sweden; ^2^ Unit of Cardiovascular and Nutritional Epidemiology Institute of Environmental Medicine Karolinska Institutet Stockholm Sweden; ^3^ Department of Surgery, Södersjukhuset Karolinska University Hospital Stockholm Sweden; ^4^ Functional Area Occupational Therapy and Physiotherapy, Allied Health Professionals Function Karolinska University Hospital Stockholm Sweden

## Abstract

**Background:**

During pregnancy, women are at risk of developing persistent symptomatic diastasis recti abdominis (DRA), which may have a detrimental effect on their physical function and quality of life (QoL). The aim of this prospective cohort study was to determine the effect of surgical repair of DRA on abdominal trunk function, urinary incontinence and QoL in postpartum women with trunk instability symptoms resistant to training.

**Methods:**

Postpartum women with diagnosed DRA and training‐resistant symptoms underwent double‐row plication of the linea alba. Abdominal trunk function was evaluated as the primary endpoint using a multimodal examination tool, the Abdominal Trunk Function Protocol. Recurrence was assessed by CT, urinary incontinence was evaluated using the Urogenital Distress Inventory (UDI‐6) and Incontinence Impact Questionnaire (IIQ‐7), and QoL was assessed with the Short Form 36 (SF‐36®) questionnaire. All subjects were examined before and 1 year after surgery.

**Results:**

Sixty women were recruited. There was no DRA recurrence at the 1‐year follow‐up. Self‐reported abdominal trunk function had improved in 98 per cent of patients, with a mean score improvement of 79·1 per cent. In the physiological tests monitored by a physiotherapist, 76 per cent performed better and endured exercise tests longer than before surgery. All SF‐36® subscales improved significantly compared with preoperative scores and reached levels similar to, or higher than, the normative Swedish female population. For the UDI‐6 and IIQ‐7, 47 and 37 per cent respectively reported fewer symptoms at follow‐up than before surgery, and 13 and 8 per cent respectively reported more symptoms.

**Conclusion:**

In this series of postpartum women presenting with DRA and symptoms of trunk instability resistant to training, surgical reconstruction resulted in a significant improvement in abdominal trunk function, urinary incontinence and QoL.

## Graphical abstract

Persistent diastasis recti abdominis after pregnancy may cause abdominal trunk dysfunctions. Surgical reconstruction improves abdominal trunk function evaluated with the novel Abdominal Trunk Function Protocol, as well as urinary incontinence and quality of life.

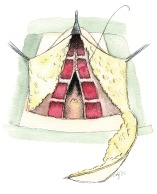

Surgical reconstruction when physical training fails

## Abstract (es)

**Antecedentes:**

Durante el embarazo, las mujeres tienen el riesgo de desarrollar una diástasis rectoabdominal persistente y sintomática (*diastasis rectoabdominis*, DRA) que puede tener un efecto perjudicial en su función física y en la calidad de vida (*quality of life*, QoL). El objetivo de este estudio de cohortes prospectivo fue determinar el efecto de reparación quirúrgica de la DRA en la función de la musculatura de la pared abdominal, la incontinencia urinaria y la QoL en mujeres con síntomas postparto de inestabilidad de la musculatura abdominal resistentes al entrenamiento.

**Métodos:**

Sesenta mujeres diagnosticadas en el postparto de DRA y con síntomas resistentes al entrenamiento se sometieron a una plicatura de doble capa de la línea alba. El objetivo primario fue evaluar la función de la musculatura de la pared abdominal mediante una herramienta de examen multimodal, el protocolo de la función de la pared abdominal (*abdominal trunk function protocol*, ATFP). La recidiva se evaluó mediante tomografía computarizada, la incontinencia urinaria utilizando los cuestionarios UDI‐6 y IIQ‐7 y la calidad de vida con el cuestionario SF‐36. Todas las participantes fueron examinadas antes de la cirugía y un año después de la misma

**Resultados:**

Tras un año de seguimiento, no hubo recidiva de la DRA. Según informaron las pacientes, la función de la musculatura abdominal había mejorado en el 98,2% de los casos, con una mejoría en la puntuación media del 79,1%. En las pruebas fisiológicas controladas por un fisioterapeuta, el 76,0% presentó mejoría y aumentó la duración en las pruebas de resistencia en comparación con antes de la cirugía. Todas las subescalas del SF‐36 mejoraron significativamente en comparación con las puntuaciones preoperatorias y alcanzaron niveles similares o superiores a los de la población femenina sueca normal. Para el UDI‐6 y el IIQ‐7, el 46,7% y el 36,7% respectivamente, manifestaron menos síntomas en el seguimiento que antes de la cirugía, mientras que el 13,3% y el 8,3% respectivamente, reportaron más síntomas.

**Conclusión:**

En esta serie de mujeres que presentan DRA en el posparto y síntomas de inestabilidad de la musculatura abdominal resistente al entrenamiento, la reconstrucción quirúrgica produjo una mejora significativa en la función de la musculatura abdominal, la incontinencia urinaria y la calidad de vida.

## Introduction

Diastasis recti abdominis (DRA) is a common and expected condition during pregnancy^1^, owing to mechanical stretching, expansion and hormonal changes^2^. The condition is often characterized by bulging or sagging in the abdominal midline during abdominal muscle contraction. Although no consensus regarding the definition of DRA exists, it is often defined in the literature as separation of the recti greater than 22–30 mm^3^, ^4^, ^5^.

DRA usually regresses to its prepregnancy width, but the condition persists in 32–46 per cent of postpartum women^6^, ^7^, ^8^, ^9^. Reported risk factors for persistent DRA include maternal age, multiparity, caesarean section, macrosomia and multiple gestations^10^. A persistent DRA may be associated with abdominal trunk instability, which could result in development of lower back pain, lack of trunk strength and urinary incontinence^11^, ^12^, ^13^. However, it remains unclear whether the DRA actually causes these symptoms or not. Although inconclusive^7^, ^8^, ^14^, persistent lower back pain after pregnancy has been reported in 11–21 per cent of postpartum women^15^, ^16^, ^17^.

The management of DRA is also a subject of discussion. Conservative management with training and weight loss is generally advised as first‐line treatment. There is no strong evidence that training during pregnancy and in the postpartum period decreases the risk of persistent DRA^18^, ^19^, ^20^, although some studies^8^, ^21^ have reported that specific exercises could increase abdominal trunk stability and reduce some of the associated symptoms. Surgical reconstruction has been reported to restore abdominal trunk function^22^, ^23^, ^24^, ^25^, ^26^ and improve lower back pain and urinary incontinence^24^, ^26^. General awareness of symptomatic DRA is poor, and patients are commonly advised to undertake non‐specific physical training, or told that the condition is only cosmetic in nature. To evaluate potential treatments for persistent symptomatic DRA, a standardized and comprehensive multimodal protocol, able to capture the wide panorama of dysfunctions associated with the condition, is required.

Thus, symptomatic persistent DRA lacks clarity of definition and management^18^, ^20^. This study aimed to evaluate the effect of surgical reconstruction of DRA in postpartum women, where no improvement in symptoms had been achieved by adequate physical training, using a standardized multimodal examination.

## Methods

Women with symptomatic DRA were recruited between January 2015 and March 2017. All first underwent ultrasound measurement of the DRA, CT to localize any concomitant ventral hernia, and assessment of trunk function by a physiotherapist. Potential candidates received an individualized trunk stabilization training programme, and were re‐evaluated by a physiotherapist after 3–6 months. Candidates presenting with subjective training‐resistant trunk symptoms after evaluation were considered eligible for inclusion. Inclusion criteria were: non‐smoker, age 18–55 years, BMI below 35 kg/m^2^, DRA greater than 30 mm on ultrasound imaging at any level, trunk instability symptoms persisting after more than 3 months of standardized trunk stability training, more than 1 year from last delivery, and no intention of further pregnancy. The presence of preoperative cosmetic issues was not considered as a symptom or as an outcome in this study. Written informed consent was obtained from all participants before inclusion. The Regional Ethics Committee, Karolinska Institutet, Stockholm, approved the study. The local ethics committee approved all procedures (Dnr. 2015/1753‐31).

### Evaluation of symptoms

The primary outcome was abdominal trunk function. Secondary outcomes were quality of life (QoL), urinary incontinence and DRA recurrence (at follow‐up).

To evaluate abdominal trunk function, a standardized multimodal trunk function test was designed to cover all dysfunctions associated with symptomatic DRA: the Abdominal Trunk Function Protocol (ATFP) (*Appendix* *S1*, supporting information). The ATFP consists of a self‐rating section, where the participants score physical function (Disability Rating Index (DRI)), and seven trunk function tests supervised and monitored by a physiotherapist following a strict schema. The validated DRI covers 12 non‐specific activities of daily life, each one self‐rated on a visual analogue scale of 0–100 mm, providing a score of 0–100 for each activity, where 0 represents no difficulty in performing the specific task and 100 indicates an inability to perform the task at all^27^. The seven trunk function tests have been validated separately and measure different aspects of trunk and pelvic strength, endurance and stability. They are: the back muscle strength test, the abdominal muscle strength test, the lateral core stability test (left and right side), the ventral core stability test, the active straight‐leg‐raising test, and the pelvic joint provocation test^28^, ^29^. The trunk function tests were conducted and monitored by a physiotherapist. The ATFP evaluation was performed before and 1 year after surgery. QoL was evaluated using the self‐reported Medical Outcome Survey Short Form 36 (SF‐36®) (Rand Corporation, Santa Monica, California, USA)^30^. Urinary incontinence was evaluated using the self‐reported Urogenital Distress Inventory (UDI‐6) and the Incontinence Impact Questionnaire (IIQ‐7) forms^31^. DRA recurrence was assessed by CT 1 year after surgery, and was defined as a persisting diastasis greater than 30 mm.

### Surgical reconstruction technique

**Figure 1:**
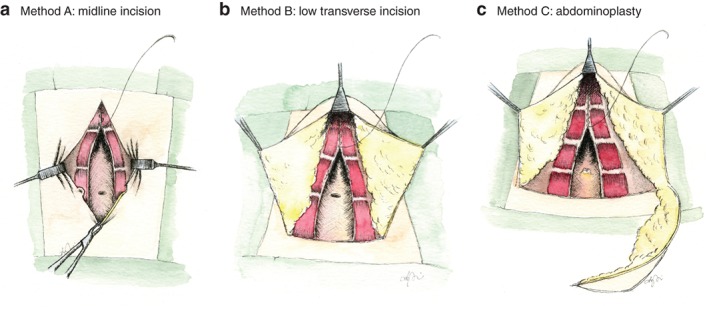
**Illustrations of the three surgical procedures** Standardized double‐layer plication of the linea alba was used without entering the rectus sheet, with absorbable self‐retaining Quill™ 2/0 suture. **a** Method A: midline incision with a suture repair and without skin excision. **b** Method B: low transverse incision with a suture repair and limited skin excision with a floating umbilicus. **c** Method C: low transverse incision with a suture repair, skin excision and umbilical transposition.

The surgical procedure was a standardized suture repair of the diastasis using a double‐row plication with absorbable Quill™ 2/0 sutures (Angiotech, Reading, Pennsylvania, USA). Access to the linea alba depended on anatomical conditions, body figure and excess skin. The surgical procedure was categorized according to the incision made: method A used a midline incision; method B involved a low transverse incision, including limited resection of excessive skin and a floating umbilicus; and method C employed abdominoplasty, including resection of excessive skin and umbilical transposition (*Fig*. 1 *a–c*). The three methods had an identical deep muscle layer technique. The decision of which method to use was based on the anatomical circumstances and the woman's preference (after detailed information regarding the risks associated with the different incisions).

All surgical procedures except one were performed by one of two consultant surgeons, at either the general surgical unit or the ambulatory surgical unit. All women were admitted to the ward for postoperative care. Patients operated on with methods B and C had an active 14‐Fr catheter drain(s) that was removed when fluid loss was less than 50 ml/day according to local routines for ventral hernia repair. All women were instructed to wear a girdle for 12 weeks (day and night in weeks 1–8, daytime only in weeks 9–12), which has been standard (with minor adjustments) in previous studies^32^. Patients were also instructed to participate in a standardized rehabilitation programme developed by the physiotherapy department at the authors' hospital (*Appendix* *S2*, supporting information), as well as daily exercise such as short walks, but to avoid heavy physical exercise during the first 12 weeks. All women were followed up clinically and with ultrasound assessment at 6–8 weeks and 1 year after surgery. All postoperative complications within 30 days were registered.

### Statistical analysis

Descriptive statistics were used to characterize demography. Pairwise correlation coefficients were performed between measurements of rectus diastasis, comparing preoperative ultrasound and CT scan measurements with the width measured at surgery. For continuous variables, paired *t* tests and Wilcoxon signed rank tests were used to identify changes in symptoms at 1‐year follow‐up, and McNemar's test was used to evaluate contingency of dichotomous variables. All tests were two‐sided and considered statistically significant at a level of *P* ≤ 0·050. For the DRI, each parameter was investigated individually and the total DRI score was used for comparison purposes. SF‐36® results were analysed and compared with data from 2679 women aged 15–44 years in the Swedish SF‐36 Health Survey^30^ (*Table* *S1*, supporting information). Linear regression was used to test whether the degrees of preoperative symptoms were associated with change in those symptoms after surgery. Non‐linearity was investigated by adding a quadratic term of the preoperative variable investigated in the model. Statistical analyses were performed using Stata® 12.1 (StataCorp, College Station, Texas, USA).

## Results

*Table* 1 summarizes the preoperative demographics of the 60 women who were included in the study. Their mean age at the time of surgery was 38·8 (range 20·5–53) years. The follow‐up rate was 93 per cent (56 of 60) for the DRI questionnaire, and 83 per cent (50 of 60) for the seven functional tests. There were four dropouts due to subacute orthopaedic surgery (1 patient), emigration (1), unrelated psychiatric disability (1) and declined further participation (1). A further six participants were excluded from follow‐up of the functional tests owing to incomplete forms.

**Table 1:** Preoperative characteristics of women who had surgery for diastasis recti abdominis

	No. of patients* (*n* = 60)
**Age (years)** †	38·8(5·5)
**BMI (kg/m** ^**2**^ **)** ‡	22·6 (17·2–36·0)
**No. of births** ‡	2 (1–5)
Vaginal delivery	2 (1–4)
Caesarean section	2 (1–4)
**Time from last birth to surgery (months)** ‡	34 (12–192)
**Duration of training before surgery (months)** ‡	7 (3–24)
**Size of diastasis recti (cm)** ‡	
Ultrasonography	4·5 (3·0–9·0)
CT	5·0 (1·0–10·0)
Perioperative finding	4·5 (3·0–9·0)
**Ventral hernia**	45 (75)

* With percentages in parentheses unless indicated otherwise; values are

† mean(s.d.) and

‡ median (range).

### Surgery

Nineteen of the 60 women (32 per cent) underwent surgical method A, 31 (52 per cent) had method B, and ten (17 per cent) method C. There was at least one concomitant midline fascial defect in 45 women (75 per cent), of which six (13 per cent) were diagnosed at surgery. The correlation coefficient between the rectus diastasis measured by ultrasonography and the intraoperative finding was 0·71. The corresponding coefficient for CT was 0·55. In general, ultrasound imaging tended to underestimate the mean diastasis by 4 mm (*P* = 0·007) and CT overestimated by 3 mm (*P* = 0·139). The median hospital stay was 3 (range 1–8) days.

### Postoperative complication and recurrence rates

At the 6–8‐week follow‐up, seven women had a postoperative complication. Four women (3 operated on with method B and 1 with method C) developed bleeding/haematoma that needed reoperation (Clavien–Dindo grade IIIb^33^, ^34^). Two patients (operated on with method B) developed a surgical‐site infection requiring antibiotic treatment (Clavien–Dindo grade II). One patient (operated on with method A) presented with spontaneous pneumothorax not requiring intervention 2 weeks after surgery (Clavien–Dindo grade I). Four patients (3 operated on with method B and 1 with method C) developed a seroma not requiring intervention, diagnosed at clinical follow‐up 6–8 weeks after surgery (Clavien–Dindo grade I). Finally, four women were not satisfied with the cosmetic result due to umbilical asymmetry, of whom two had reoperation; this was not considered a complication. None of the early complications had led to long‐term sequelae at the 1‐year follow‐up. Complications according to the Clavien–Dindo classification^33^, ^34^ were in summary: grade I, five of 60 (8 per cent); grade II, two of 60 (3 per cent); grade IIIb, four of 60 (7 per cent). No recurrences were observed at 6–8 weeks or at 1‐year follow‐up.

### Abdominal Trunk Function Protocol

**Figure 2:**
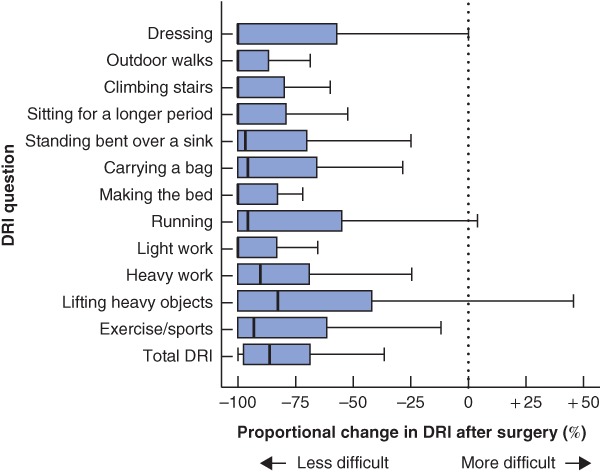
Box plot of the proportional change in the Disability Rating Index score at 1 year *versus* before surgery Median values and interquartile ranges are denoted by horizontal bars and boxes respectively; error bars have been drawn to span all data points within 1·5 i.q.r. of the nearer quartile. Outliers have been excluded. DRI, Disability Rating Index.

**Figure 3:**
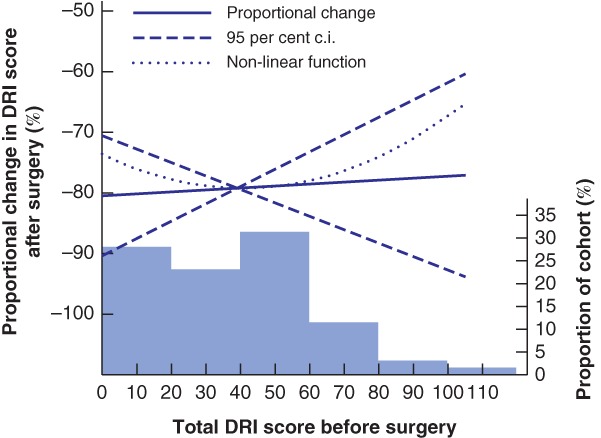
Proportional change in Disability Rating Index score after surgery Proportional change in mean total Disability Rating Index (DRI) score after surgery as a function of the preoperative score in a linear regression model. *P*_non‐linearity_ = 0·740. The histogram represents the preoperative score distribution.

**Figure 4:**
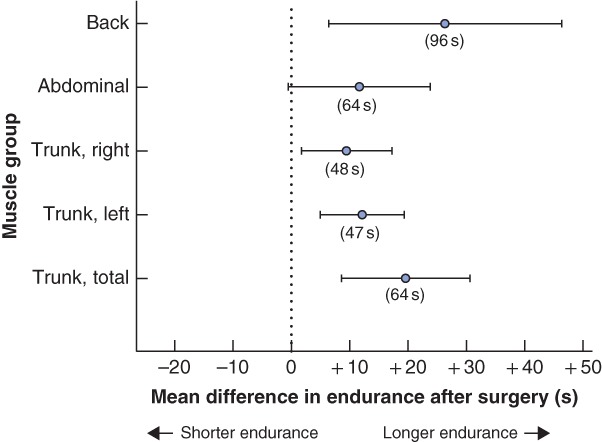
Mean change in endurance of the various physical tests before and after surgery Tests were standardized and evaluated by a physiotherapist. Values in parentheses represent the mean score of each variable before surgery.

*Table* 2 summarizes the ATFP findings before surgery and at 1‐year follow‐up. Regarding the DRI, 98 per cent of women (55 of 56) reported fewer problems after surgery, and the total scores were, on average, 79·1 (95 per cent c.i. 73·1 to 85·1) per cent lower at follow‐up than before surgery. One patient reported a higher score after surgery (total DRI 98 before surgery *versus* 105 after surgery). Median scores and proportional change after surgery for each specific question are displayed in *Fig*. 2. The preoperative score was not associated with proportional change in DRI at follow‐up (*P* = 0·804) (*Fig*. 3). When evaluated by a physiotherapist, a majority of patients (38 of 50, 76 per cent) had significantly better performance and stamina at follow‐up than before surgery. There was no significant change in pain and pelvic tip during straight‐leg raising. Although a significant proportion of the women performed better in the postoperative tests, the mean abdominal strength did not appear to have improved at 1‐year follow‐up (*Fig*. 4).

**Table 2:** Abdominal Trunk Function Protocol, Urogenital Distress Inventory and Incontinence Impact Questionnaire results before and 1 year after surgery for diastasis recti abdominis

	Before surgery* (*n* = 60)	After surgery* (*n* = 60)	*P* #
**Abdominal Trunk Function Protocol**			
Specific DRI (0–100 points)†, §			
Dressing	1 (0·0–7·0)	0 (0·0–1·0)	0·006
Outdoor walks	4 (0·0–9·5)	0 (0·0–4·4)	< 0·001
Climbing stairs	3 (0·0–9·4)	0 (0·0–4·4)	< 0·001
Sitting for a longer period	28 (0·0–9·6)	0 (0·0–5·2)	< 0·001
Standing bent over a sink	41 (0·0–10·0)	0 (0·0–5·3)	< 0·001
Carrying a bag	29 (0·0–8·3)	1 (0·0–4·9)	< 0·001
Making the bed	13 (0·0–8·4)	0 (0·0–4·5)	< 0·001
Running	49 (0·0–10·0)	1 (0·0–9·6)	< 0·001
Light work	21 (0·0–10·0)	0 (0·0–5·0)	< 0·001
Heavy work	64 (0·0–10·0)	5 (0·0–9·8)	< 0·001
Lifting heavy objects	63 (0·1–10·0)	9 (0·0–9·8)	< 0·001
Exercise/sports	54 (0·1–10·0)	5 (0·0–9·4)	< 0·001
Total DRI score (0–120 points)‡	386(247)	82(118)	< 0·001**
Physiological tests¶			
Back muscle strength (s)†	75 (0–240)	113 (0–240)	< 0·001
Abdominal muscle strength (s)†	49 (0–240)	66 (15–240)	< 0·001
Core stability, side plank (s)†	40 (0–120)	56 (10–115)	< 0·001
Core muscle strength and stability test (s)†	60 (0–180)	74 (3–180)	0·004
Difficulties with active straight leg raising (1–5 points)†	1 (1–5)	1 (0–2)	< 0·001
Pain during straight leg raising	8 (13)	3 (5)	0·096††
Pelvic tip during straight leg raising	9 (15)	9 (15)	1·000††
Pain during pelvic provocation	12 (20)	3 (5)	0·020††
**Urogenital Distress Inventory (UDI‐6)** †	5 (0–16)	2 (0–13)	0·001
**Incontinence Impact Questionnaire (IIQ‐7)** †	2 (0–18)	0 (0–17)	0·002

* With percentages in parentheses unless indicated otherwise; values are

† median (range) and

‡ mean(s.d.).

§ The Disability Rating Index (DRI) was standardized and recorded on visual analogue scales (measured in millimetres), providing a score with a range of 0–100 for each activity where 0 represented no difficulty at all in performing the specified task and 100 indicated not being able to perform the task at all).

¶ Physiological tests were conducted and monitored by a physiotherapist.

# Wilcoxon signed rank test, except

** paired *t* test and

†† McNemar test.

### Quality of life

**Figure 5:**
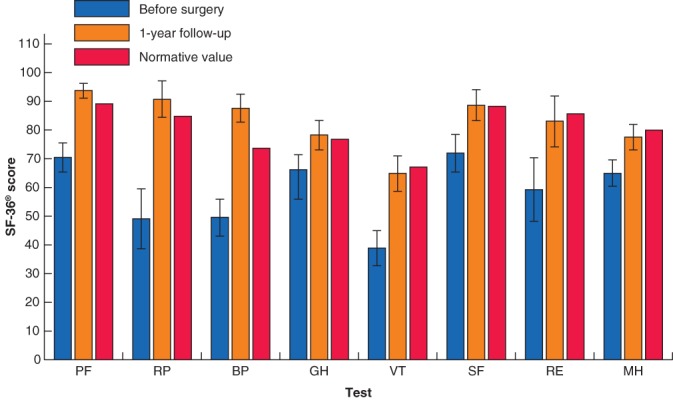
Bar chart showing Medical Outcome Survey Short Form 36 (SF‐36®) scores for patients with symptomatic diastasis recti abdominis Mean SF‐36® scores before and 1 year after surgery are compared with normative values from 3994 women aged 15–64 years in the Swedish SF‐36 Health Survey. Error bars indicate 95 per cent confidence intervals. PF, physical function; RP, role physical; BP, bodily pain; GH, general health; VT, vitality; SF, social functioning; RE, role emotional; MH, mental health.

Mean SF‐36® subscale scores, comparing results before and after surgery, and with expected ratings in a normative Swedish female population, are shown in *Fig*. 5. Before surgery, the women generally had a lower QoL than the normative Swedish female population in all SF‐36® subscales (*P* < 0·003). After surgery, their QoL improved significantly, with scores similar to those of the normative Swedish female population in all subscales, and even higher in terms of bodily pain (*P* < 0·001).

### Urinary incontinence

**Figure 6:**
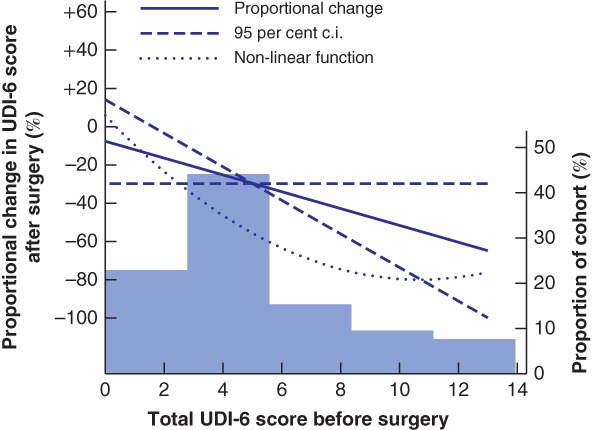
Proportional change in Urogenital Distress Inventory score after surgery Proportional change in mean total Urogenital Distress Inventory (UDI‐6) score after surgery as a function of the preoperative score in a linear regression model. *P*_non‐linearity_ = 0·062. The histogram represents the preoperative score distribution.

*Table* 2 summarizes urinary incontinence before surgery and at 1‐year follow‐up. A general decrease in incontinence symptoms was observed after surgery, with 28 of the 60 women (47 per cent) reporting a lower score in the UDI‐6 after surgery, and eight (13 per cent) reporting a higher score. The mean reduction in score after surgery was 34·4 (95 per cent c.i. 16·4 to 52·3) per cent. There appeared to be an inverse linear relationship between scores before and after surgery, with each point scored before surgery related to a 0·39‐point decrease in UDI‐6 after surgery. Although no formal evidence of non‐linearity was observed (*P* = 0·062), no relief of symptoms was observed after surgery when 2 or fewer points were scored before surgery. Otherwise, the proportional decrease in UDI‐6 score remained between 40 and 80 per cent (*Fig*. 6).

For the IIQ‐7, 22 of the 60 women (37 per cent) had fewer symptoms and five (8 per cent) experienced more symptoms.

## Discussion

DRA is a potentially debilitating condition in postpartum women that correlates with trunk instability, urinary incontinence and impaired QoL. In this study, a significant improvement in self‐reported disability and physical performance, higher QoL and reduced urinary incontinence was observed in the majority of women after surgical treatment of DRA. A novel multimodal protocol to evaluate abdominal trunk function was also introduced.

All women reported a better QoL (SF‐36® findings) after surgery, reaching levels similar to those in a normative Swedish female population, regardless of performed surgical method. This indicates that the selection process was successful in distinguishing between physical and cosmetic reasons for surgery. The ATFP focused on functional disability that was resistant to training, thereby selecting patients likely to benefit from surgery. The fact that proportional improvements in DRI and physical tests were similar for all patients, regardless of preoperative scores, implies that surgical reconstruction leads to improvement in function in all postpartum women with DRA. With respect to the postoperative rehabilitation programme, it is not likely that rehabilitation with a lower load than the preoperative training would have had any significant impact on the improvement.

It is suggested that patients with DRA not causing dysfunction (DRA less than 30 mm, midline hernia or cosmetic issues) should first and foremost receive conservative management with weight control, limited hernia repair or purely aesthetic surgery. Evidence in the literature supporting physical training for symptomatic DRA^18^, ^19^, ^20^, ^21^ is inconclusive. The main purpose of physical training is possibly to restore function, and not necessarily to reduce the diastasis. If physical training proves to be unsuccessful, surgical reconstruction may be the next step in the treatment algorithm.

The improvement in urinary incontinence symptoms observed in this study is in line with previous studies^26^, and may indicate a correlation between abdominal trunk instability and pelvic floor dysfunction. Higher preoperative UDI‐6 scores resulted in greater improvements in urinary incontinence symptoms after surgery, suggesting that patients with severe symptoms benefit more from surgical reconstruction than those with mild symptoms – an important factor when selecting patients for surgery.

All three surgical techniques used in this study provided similar results regarding outcome and recurrence. None of the women had signs of recurrence 1 year after surgery, and the complication rate was similar to that following other medium‐sized surgery such as open ventral hernia repair. There were no differences in QoL outcomes between the three surgical methods, indicating that cosmetic improvement alone was unlikely to be the reason for the improvement in QoL.

The high incidence of perioperative ventral hernia in the study sample could indicate that these hernias contributed to the symptoms; however, the presenting symptoms are not usually associated with a ventral hernia and it is unlikely that the concurrent hernia repair alone could explain the results.

This study has limitations. It lacked a conservatively managed control group, which makes any far‐reaching conclusion difficult as some beneficial effects could have been a placebo effect or simply due to the passing of time, although comparison of preoperative and postoperative results allows within‐person changes to be measured. The inclusion criteria were restricted to patients with symptomatic persistent DRA, and these results are thus not applicable to all patients with postpartum DRA.

During pregnancy, women are at risk of developing persistent symptomatic DRA that may have a detrimental effect on their physical function and QoL. This study has demonstrated that surgical reconstruction of DRA in postpartum women with symptoms resistant to training results in significant improvements in abdominal trunk function, urinary incontinence and QoL for a majority of patients. Surgical reconstruction of DRA is a valid alternative for patients presenting with symptomatic DRA, where adequate physical training has proven unsuccessful.

## Supporting information


**Appendix S1** The Abdominal Trunk Function Protocol (ATFP), including the Disability Rating Index (DRI) and the seven function tests
**Appendix S2** The local standardized rehabilitation programme
**Table S1** Quality of life before surgery, at 1‐year follow‐up, and among 3994 women aged 15–64 years in the Swedish SF‐36 Health Survey
