# Supplementary material for: Cohort study of the effect of surgical repair of symptomatic diastasis recti abdominis on abdominal trunk function and quality of life
Source: BJS Open. 2019 Sep 11;3(6):750–8. doi: 10.1002/bjs5.50213 (PMC6887686; doi:10.1002/bjs5.50213)
Supplement: Supplementary file 1 — Appendix S1 The Abdominal Trunk Function Protocol (ATFP), including the Disability Rating Index (DRI) and the seven function tests Appendix S2 The local standardized rehabilitation programme Table S1 Quality of life before surgery, at 1‐year follow‐up, and among 3994 women aged 15–64 years in the Swedish SF‐36 Health Survey [file BJS5-3-750-s001.docx]

**BJS5_50213**

**Cohort study of the effect of surgical repair of symptomatic diastasis recti abdominis on abdominal trunk function and quality of life**

**A. Olsson, O. Kiwanuka, S. Wilhelmsson, G. Sandblom and O. Stackelberg**

**Appendix S1** The Abdominal Trunk Function Protocol (ATFP), including the Disability Rating Index (DRI) and the seven function tests














**Appendix S2** The local standardized rehabilitation programme








**Table S1** Quality of life before surgery, at 1-year follow-up, and among 3994 women aged 15–64 years in the Swedish SF-36 Health Survey
